# A Mathematical Model of Tumor Volume Changes during Radiotherapy

**DOI:** 10.1155/2013/181070

**Published:** 2013-10-03

**Authors:** Ping Wang, Yuanming Feng

**Affiliations:** ^1^Department of Radiation Oncology, Tianjin Medical University Cancer Institute and Hospital, Tianjin 300060, China; ^2^Departments of Radiation Oncology and Physics, East Carolina University, Greenville, NC 27834, USA

## Abstract

*Purpose*. To develop a clinically viable mathematical model that quantitatively predicts tumor volume change during radiotherapy in order to provide treatment response assessment for prognosis, treatment plan optimization, and adaptation. *Method and Materials*. The correction factors containing hypoxia, DNA single strand breaks, potentially lethal damage, and other factors were used to develop an improved cell survival model based on the popular linear-quadratic model of cell survival in radiotherapy. The four-level cell population model proposed by Chvetsov et al. was further simplified by removing the initial hypoxic fraction and reoxygenation parameter, which are hard to obtain in routine clinics, such that an easy-to-use model can be developed for clinical applications. The new model was validated with data of nine lung and cervical cancer patients. *Results*. Out of the nine cases, the new model can predict tumor volume change in six cases with a correlation index *R* ^2^ greater than 0.9 and the rest of three with *R* ^2^ greater than 0.85. *Conclusion*. Based on a four-level cell population model, a more practical and simplified cell survival curve was proposed to model the tumor volume changes during radiotherapy. Validation study with patient data demonstrated feasibility and clinical usefulness of the new model in predicting tumor volume change in radiotherapy.

## 1. Introduction

As the information from posttherapy imaging usually comes too late to realistically impact patient management, early prediction of therapy outcome is of paramount importance [1, 2]. Many literatures have confirmed that therapy-induced tumor volume changes can be used to predict ultimate local tumor control and patient survival [1–3]. This leads to increased research interests in monitoring response of the tumor volume to radiotherapy.

A substantial number of mathematical models for the analysis of the tumor volume change have been developed based on clinical data and the linear-quadratic (LQ) model. These models span from simple tumor-volume models similar to those proposed by Fischer [4–6] to more complicated computer implementations that are based on 3D individual cell simulations using random processes simulated with Monte Carlo methods [7, 8]. But they do not use underlying radiobiologic mechanisms [9].

As the development of functional and molecular imaging techniques and equipment over recent years, such as functional magnetic resonance imaging (fMRI) and positron emission tomography (PET), radiobiologic tumor regression models have been studied. The four-level population model proposed by Chvetsov et al. combines cell hypoxia, reoxygenation, proliferation, disintegration, and other radiobiologic phenomena, which shows meaningful breakthrough and more innovation than previous studies [9]. These approximations improve accuracy of the model. But they are difficult to be used in clinical settings. For instance, measurements of initial hypoxic fraction and reoxygenation rate of tumors have not been implemented in clinical cancer management because of the high cost burden on patients.

Improvement to the LG model is needed to extend its applicability in radiobiology studies. Wang et al. reported that the model took into account only the repair process, whereas the reduction of sublethal lesions owing to conversion to lethal lesions with further radiation had been ignored. This is acceptable in low fraction dose and low-dose rate (LDR) irradiation; however, it could lead to large discrepancies in high fraction dose and high-dose rate (HDR) irradiation [4].

Therefore, an improved cell survival model based on the conventional LQ model and simplified cell population model is needed to promote the development of personalized therapy. The aim of this paper is to develop a clinically viable mathematical model that quantitatively predicts tumor volume change during radiotherapy in order to provide treatment response assessment for prognosis, treatment plan optimization, and adaptation.

## 2. Methods and Materials

### 2.1. Cell Survival Curve

According to the linear-quadratic model in radiation biology, the average number of DNA double strand breaks (*N*) by ionizing radiation in a single cell is assumed to be a linear-quadratic function of radiation dose (*D*) as shown in the following:

(1) $$\begin{array}{c} N=\alpha \times D+\beta \times D^{2}. \end{array}$$

It assumes that there are “two” components involved in cell killing: the linear component (*α*), which characterizes a single lethal event made by one track action, and the quadratic component (*β*), which characterizes the accumulation of sublethal events made by two-track action [10]. As such, the conventional cell survival curve can be expressed by the following [11, 12]:

(2) $$\begin{array}{c} S=e^{-N}=e^{-(\alpha D+\beta D^{2})}. \end{array}$$

However, this cell survival equation is based on series of hypotheses and assumptions, which do not reflect and explicate all events of DNA damage induced by ionization.

1. Both types of events describe the DNA double strand breaks, whereas the damage caused by the DNA single strand breaks has been ignored. Studies confirmed that X-ray induced base damage and single-strand damage that are far more than the number of double-strand damage, often 50 times more, and, under certain conditions, the DNA single-strand damage can be converted into double-strand damage resulting in the stop of cell proliferation [11].
2. The definition of reproductive death is the loss of the capacity for sustained proliferation, that is, the loss of reproductive integrity. This definition reflects a narrow view of radiobiology. A cell may be physically present and apparently intact, may be able to make proteins or synthesize DNA, and may even be able to struggle through one or two mitoses, but if it has lost the capacity to divide indefinitely and produce a large number of progeny, it is by definition dead [11, 12]. However, this kind of tumor cells and their colonies, which may have a large impact with their portion increasing continuously during treatment, have not been taken into account when calculating the tumor volume.
3. Radiation exposure can alter the environment surrounding cells so that cells may be potentially lethal damaged (PLD). It has been suggested that capability of PLD repair plays an important role in the resistance to treatment for human cancers. Such that, cell survival ratio may be increased to exceed or be reduced to less than the nominal value by the effect of specific dose. Therefore, the overall level of *N* will be affected.
4. Researchers have found that the cell's oxygen and blood supply have an impact on the tumor radiosensitivity. So, the microenvironment of cancer cells will affect the overall level of *N*.

To take these factors into account, we proposed two correction factors, *A* and *B*, to make an improvement to the cell survival model. The new model is defined as

(3) $$\begin{array}{c} S=e^{-A\times (\alpha D+\beta D^{2})+B}, \end{array}$$

where *A* represents a variety of undefined factors related to cancer staging, histological grade, oxygen supply, blood flow, and the living environment after irradiation *B* represents the impact caused by the events that are not included in *α* and *β* events, such as DNA single strand break and cell proliferation before reproductive death.

This modification takes more cancer-specific factors into account and enables a better expression to the real situation. In addition, cell hypoxia factor is also taken into account, which lays a foundation for the simplification of the four-level cell population model which will be presented in the next section.

### 2.2. Model of Tumor Volume Change

The four-level population model proposed by Chvetsov et al. is one of the comprehensive models. But the measurement of initial hypoxic fraction and reoxygenation rate of the tumor makes it difficult for clinical application. So we considered simplifing the model by transferring the initial hypoxic fraction and reoxygenation parameter to the improved cell survival curve, such that an easy-to-use model can be developed for clinical applications.

The tumor volume (*V*) is assumed to be proportional to the total number of cells in the tumor (*N*_*t*_); it can be evaluated using the following equation:

(4) $$\begin{array}{c} V=N_{t}\times v, \end{array}$$

where *v* is a constant that includes the volume of a single cell and the volume of the related intercellular space. Radiation will cause death of some proliferative cells. After irradiation with dose *D*, the initial total number of cells in the tumor (*N*_*t*,0_) is changed to

(5) $$\begin{array}{c} {\bar{N}}_{l,1}=N_{t,0}\times S,\\ {\bar{N}}_{d,1}=N_{t,0}\times \left(1-S\right). \end{array}$$

${\bar{N}}_{l,1}$ and ${\bar{N}}_{d,1}$ are the number of viable and lethally damaged cells after first irradiation. The number of cells after the *k*th dose fraction is

(6) $$\begin{array}{c} {\bar{N}}_{l,k}=N_{l,k-1}\times S,\\ {\bar{N}}_{d,k}=N_{d,k-1}+N_{l,k-1}\times \left(1-S\right). \end{array}$$

During the time interval (Δ*t*_*k*_) between *k* and (*k* + 1) dose fraction, the total number of cells in the tumor changes as a result of the proliferation of dividing viable cells and the disintegration of lethally damaged cells. The repopulation of viable cells is governed by its exponential growth with the constant *λ* as shown in the following:

(7) $$\begin{array}{c} \lambda =\frac{\ln2}{T_{\text{pot}}} \end{array}$$

which is related to the potential doubling time (*T*_pot_). The disintegration of lethally damaged cells is modeled using the exponential decay with the decay constant *μ*:

(8) $$\begin{array}{c} \mu =\frac{\ln2}{T_{1/2}}, \end{array}$$

where *T*_1/2_ is the volume halving time.

Taking the models for proliferation and disintegration into account, the cell number will change during the time interval between dose fractions and can be expressed as follows:

(9) $$\begin{array}{c} N_{l,k}={\bar{N}}_{l,k}\exp\left(\lambda \Delta t_{k}\right),\\ N_{d,k}={\bar{N}}_{d,k}\exp\left(-\mu \Delta t_{k}\right). \end{array}$$

And the total number of cells and the tumor volume at time of *t* and after the *k*th dose fraction are

(10) $$\begin{array}{c} N_{t,k}=N_{d,k}+N_{l,k},\\ V_{t,k}=N_{t,k}\times v. \end{array}$$

### 2.3. Model Validation

MATLAB R2010a (The Math Works, Inc., Natick, USA) and CT image data of nine patients (four lung cancer cases and five cervical cancer cases) were used to validate the new model in comparison with the results from the conventional model. The patients received radiotherapy treatment, and three to four sequential CT image sets were acquired on day one and in the interval of about one to two weeks during the treatment. Tumors were delineated by an experienced radiation oncologist on the CT images, and volumes were calculated accordingly [13]. The initial volume (*V*_0_) and volume halving time (*T*_1/2_) are listed in Table 1. The parameter *T*_pot_ was obtained from published data, for the lung tumors *T*_pot_ = 5.5 days [9] and for the cervical tumors *T*_pot_ = 4.5 days [11].

The correlation index (*R* ^2^) was used to quantify the goodness of fit of the models to the actual patient data. *R* ^2^ is defined in the following:

(11) $$\begin{array}{c} R^{2}=1-\frac{\sum {\left(y-\hat{y}\right)}^{2}}{\sum {\left(y-\bar{y}\right)}^{2}}, \end{array}$$

where *y* is the measured value, $\hat{y}$ is the predicted value by the model and $\bar{y}$ is the mean of the measured data. It is generally believed that, when *R* ^2^ is greater than 0.8, a high degree of correlation exists [14].

## 3. Results and Discussion

The correlation coefficient (*R* ^2^), *A*, and *B* were obtained by using least square method. Results are shown in Table 2. Here, *R* ^2^ is the correlation coefficient between the predicted tumor volume change by using the new simplified four-level cell population model and the measured ones, and *R*_0_^2^ is the correlation coefficient between the predicted tumor volume change by using the conventional model and the measured data for comparison.  Figure 2 shows the measured data and the modeled results of the tumor volumes for the 9 cases.

**Figure 1:**
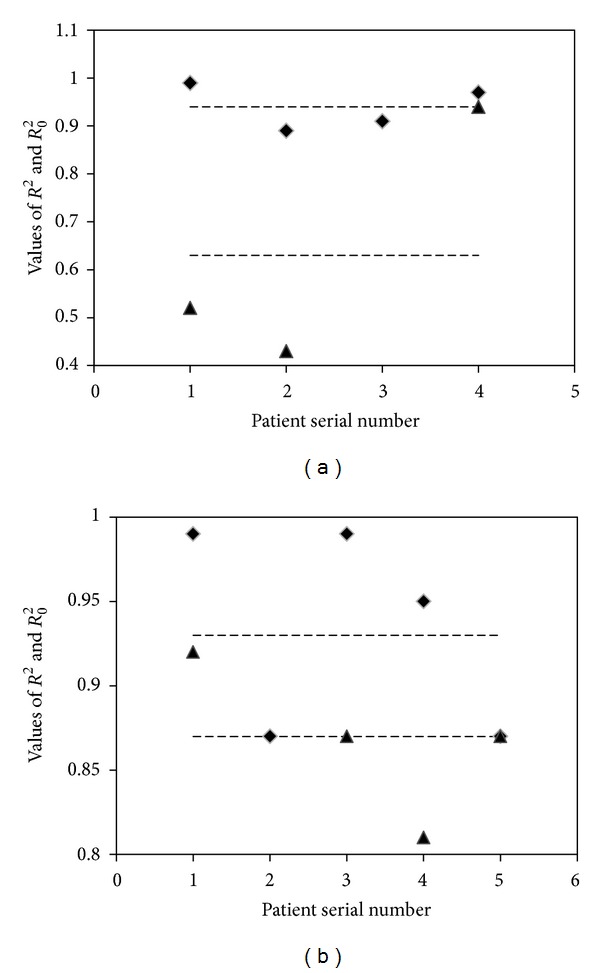
Correlation coefficients of the models: diamonds: *R* ^2^; triangles: *R*_0_^2^; dash lines: mean values. (a) Lung cancer cases, with mean value of 0.94 for *R* ^2^ and 0.63 for *R*_0_^2^, respectively; (b) cervical cancer cases, with mean value of 0.93 for *R* ^2^ and 0.87 for *R*_0_^2^, respectively.

**Figure 2:**
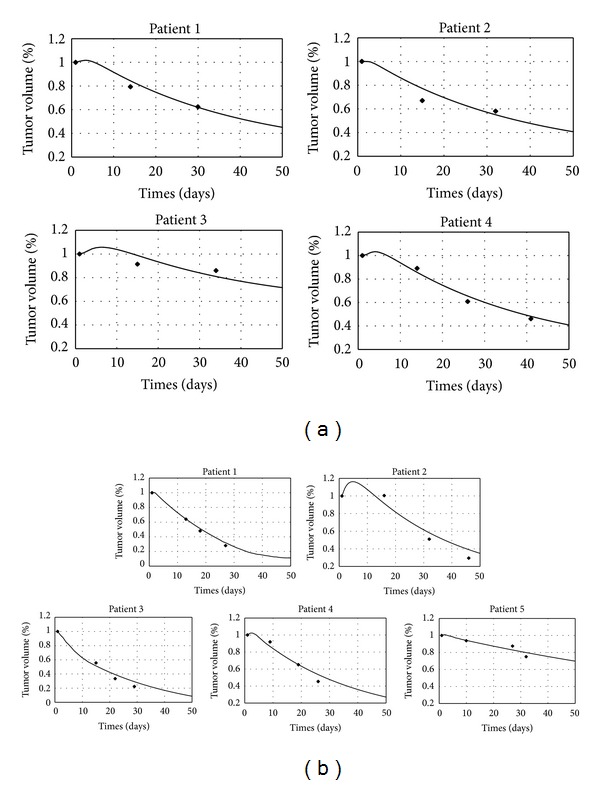
Measured (symbols) and modeled results (solid lines) of the tumor volumes. (a) Lung cancer cases, (b) cervical cancer cases.

From Table 2, it can be seen that the new model can predict the tumor volume change in six of the nine cases with correlation index *R* ^2^ greater than 0.9 and the rest of three cases with *R* ^2^ greater than 0.85. When comparing the values of *R* ^2^ with *R*_0_^2^ in Figure 1, it is clear that *R* ^2^ is greater than *R*_0_^2^ for all the cases and the mean values of *R* ^2^ is significantly greater than the ones of *R*_0_^2^ (0.94 > 0.63 for lung cancer cases and 0.93 > 0.87 for cervical cancer cases). It is shown that the improved cell survival model yields better prediction for tumor volume change during the radiation treatment.

The correction factor *A* is smaller than 1, which may be due to the impact of reduced amount of the total of the DNA double strand breaks caused by the PLD and microenvironment change for the irradiated cells; for example, hypoxic may increase the tumor radioresistance. *B* is negative for all the cases; this may be due to the impact from the events of neither *α* nor *β*; for example, DNA single strand break would increase cell reproductive death. The results have demonstrated the advantages of the proposed tumor volume change model and shown its feasibility and practical usefulness.

## 4. Conclusions

Several hard-to-get factors in clinical radiotherapy were considered and used in the development of a new cell survival model. The four-level cell population model was simplified to obtain an easy-to-use model for clinical applications. The combination of the new models contains more radiobiological factors, which can be used to describe tumor volume variation during the fractionated radiotherapy. As the tumor volume changes during radiotherapy could affect the received dose in the intensity-modulated radiotherapy (IMRT), the new mode proposed in this study provides a method in understanding the radiobiological processes and potential application in improving the IMRT treatments. It should be noted that certain limitations exist in the new model. For instance, *R* ^2^, *A*, and *B* were obtained by using least square method only. Future studies should take patient age, gender, tumor location, shape, stage, degree of differentiation, and other information into account, so that a more comprehensive model can be developed and used to the prediction and monitoring of the tumor volume changes during radiotherapy.

## Figures and Tables

**Table 1a:** (a) Lung cancer cases

	Patient serial number				Mean value
	1	2	3	4	
*V*_0_/(cm^3^)	7.6	27.4	97.7	189.3	80.5
*T*_1/2_/(day)	42.5	35.9	50.2	33.3	40.5

**Table 1b:** (b) Cervical cancer cases

	Patient serial number					Mean value
	1	2	3	4	5	
*V*_0_/(cm^3^)	8.0	14.2	22.1	76.1	375.2	99.1
*T*_1/2_/(day)	13.9	90.6	15.6	24.4	31.0	35.1

**Table 2a:** (a) Lung cancer cases

	Patient serial number			
	1	2	3	4
*A*	0.85	0.82	0.66	0.82
*B*	−2.03	−2.10	−2.01	−0.41
*R* ^2^	0.99	0.89	0.91	0.97
*R*_0_^2^	0.52	0.43	−0.68	0.94

**Table 2b:** (b) Cervical cancer cases

	Patient serial number				
	1	2	3	4	5
*A*	0.43	0.19	0.24	0.74	0.17
*B*	−1.66	−2.95	−1.78	−0.71	−0.63
*R* ^2^	0.99	0.87	0.99	0.95	0.87
*R*_0_^2^	0.92	−1.43	0.87	0.81	0.87
